# Editorialets

**Published:** 1908-11

**Authors:** 


### Editorialets.

For Sale at a Bargain.—The office equipment of the late Dr. J. A. Armstrong, City Physician of San Marcos, Texas, consisting of fifty-six medical and surgical books, and a large glass front book case; a miscellaneous lot of surgical instruments; one static machine, one ozone inhaler; one leather-covered operating chair. All is first-class quality and in good condition. A great bargain will be given if early application is made. Address, Albert S. Armstrong, San Marcos, Texas.

Barnes Medical College Vindicated.—The Missouri State Board of Medical Examiners last summer refused to examine graduates of Barnes Medical College, holding that it, together with others, did not come up to the minimum requirements in the matter of equipment. Twenty-eight of its alumni applied to the courts for a mandamus to compel the Board to examine them. The writ was granted, the court finding that the act of the Board was not justified by the facts.

---

The "Rigid Os," so often met with in obstetrical cases, is most effectively relieved and much suffering avoided by the administration of Hayden's Viburnum Compound. No less an authority than Dr. Sims used and recommended this standard product.

---

"The Phenomenal Lafayette."—Latest—October 31st. Suit has been filed by the county attorney at San Antonio to revoke the license of Dr. J. Lafayette Berry, alias "The Phenomenal Lafayette," for gross unprofessional conduct and "advertising calculated to deceive the public."

We shall see now if the law will "hold water."

---

The Fifth District (San Antonio) Medical Association will hold its regular semi-annual meeting November 20th (inst.) Professors Dock and Dyer, of Tulane, will be guests, and will deliver addresses.

